# The Clinical Society of Maryland

**Published:** 1901-04

**Authors:** H. O. Reik

**Affiliations:** 5 W. Preston St.; Baltimore


### SOCIETY REPORTS.

### THE CLINICAL SOCIETY OF MARYLAND.

BALTIMORE, February 15, 1901.

The meeting was held at the College of Physicians and Surgeons, and was called to order by the president, Dr. W. J. Todd.

### A CASE OF ACCESSORY THYROID GLAND.—DR. JOHN R. WINSLOW.

I have the privilege of exhibiting what I believe to be a rare growth at the base of the tongue. It answers in every way to the description of cases of accessory thyroid gland at the base of the tongue, and I believe there are only 12 or 13 such cases reported in medical literature. The patient will be brought in, and any gentleman interested in the matter may examine her.

### A MASTOID OPERATION WITH HEALING UNDER BLOOD-CLOT. EXHIBITION OF PATIENT.—DR. H. FRIEDENWALD.

The patient I want to show you is one who has interested us in many ways since he came into the hospital on the 11th of December. He had then had a fever for a week, with a temperature ranging from 101 to 102.5. He had some slight râles with pain in the chest and Dr. Lockwood though he was going to develop a pneumonia. An eruption appeared over the body, and in a very few days the diagnosis made itself clear as one of syphilitic fever; he still presents many of the marks on his body. His temperature remained above 101 for a week, and then, under specific treatment, it returned to normal and the patient seemed to be getting well. At the end of the second week he again complained of severe pain in the head, at first around the mastoid region, and the possibility of mastoiditis was considered. I should have mentioned that the patient had a double purulent otitis, the duration of which was not known. His temperature rose to 105.5, and during the next few days frequently reached that point. The pain was very definitely located in the right mastoid region, but the sensitiveness was so diffuse that we hesitated about an operation and awaited developments. Two days later they occurred in the form of a very extensive erysipelas, which lasted for two weeks.

Even when he got well of the erysipelas, however, his clinical history did not end, for after several days of normal temperature he again began to complain of intense pain in the right mastoid region, and the diagnosis of mastoiditis was very evident. There was some slight swelling, with tenderness, definitely located about the antrum and neighborhood of the tip. A mastoid operation was performed, the bone found very much sclerosed, and it was evident this was not a recent otitis, for we never find such a marked degree of sclerosis in fresh cases of otitis, and it always indicates a long-continued process. We found pus in considerable quantity, both at the tip and in the cells near the antrum. The parts were thoroughly cleaned and irrigated with a normal salt solution, and in order to try in this case again the healing under blood-clot, even under unfavorable circumstances, we allowed the wound to fill up with blood and sewed up the soft parts. At the second dressing there were a few points where there was a little suppuration, evidently stitch abscesses, and at the end of two weeks the patient had entirely recovered.

DISCUSSION.

*Dr. Cone:* I would like to ask whether, in healing up under blood-clot, any special precautions were taken to get rid of any pus that might be present, but invisible, before the wound was allowed to fill with blood.

*Dr. Friedenwald:* Only mechanical means. The wound was mopped out and washed with normal salt solution; no antiseptics were used.

*Dr. Cone:* This case is a very interesting one to consider in connection with other work that has been done to secure healing under blood-clot, for instance, in osteomyelitis. We have always felt in these cases that we had to treat the wound very thoroughly before allowing it to fill with blood, in order to increase the chances of perprimum healing. This case shows that you can get a wound to heal in a very short time under simple blood-clot. I recently had a case of healing of the tibia under blood-clot in one week, but I swabbed out with bichloride and then washed with normal salt.

*Dr. Cohen:* Was this wound sewed up without any drainage?

*Dr. Friedenwald:* Yes.

*Dr. Reik:* Dr. Friedenwald has secured a very excellent result in this case, and it illustrates very well the value of the blood-clot method. I have used it now in six cases, and in four of these the wound was perfectly healed and the patients discharged at the end of a week. One case showed a partial breaking down of the clot which drained from the lower part of the wound, and the latter healed with but slight delay, while in the other case the clot broke down completely. When successful, we are able to discharge such patients in a week or ten days, whereas if the wound be packed or drained, it requires three weeks or more to secure healing. Should the clot break down, as it did in one of my cases, the stitches may be easily removed, the wound cleaned and packed, and the patient is no worse off than if this had been done at the time of the first dressing. It certainly seems desirable to give this method much wider trial.

*Dr. Pancoast:* Were any cultures taken in any of these cases reported by Drs. Friedenwald and Reik?

*Dr. Friedenwald*: Cultures were taken in this case but I am not able to say at present what was found.

*Dr. Reik:* In three of my cases the staphylococcus was found, in one the streptococcus, and I believe no growth was obtained in the other two.

*Dr. Bernstein:* It strikes me that we are running great risks to allow a wound of that sort to heal up by blood-clot. Occasionally you will get perfect union, but every one knows the perfect culture medium that blood-clot makes, and when you have the pneumococcus, the staphylococcus or the streptococcus in the wound, the risk is great. The operation I have done, and which Koerner and Jansen do, gives quite as brilliant results, and it strikes me it eliminates that very large element of danger, the risk of general infection, from this beautiful medium. The operation is to entirely clean out the bone, do the radical operation and then perform the flap operation and drain the ear from the external auditory canal. You sew up the post-auricular wound immediately, and in the case I had in the Maryland University Hospital recently, the patient was up and about on the 5th day and discharged on the 7th day. In that case the hearing before the operation was so bad that you had to yell at him, and after the operation he could hear whispered sounds at six feet.

This operation has been universally successful and Jansen, told me last summer that he did not know what a loss meant.

*Dr. Friedenwald:* I would like to say with reference to the Koerner operation that, while it is an excellent operation in the chronic cases where you wish to clean out the whole antrum and middle ear, it is not indicated in such cases as the one described. It is certainly a very much more radical operation than the simple Schwartze operation, and you may destroy some of the functionating parts of the middle ear in doing it. In the typical mastoid operation you simply open and clean out that part of the mastoid which is infected.

The danger can not be very great, for the operation of healing under blood-clot has been tried now in quite a large number of cases by Blake of Boston, and others, and in Blake's hands its success has been really astounding. It was after reading his report that I tried it, and with the most satisfactory results in this and another case.

A CASE OF GASTROSTOMY. EXHIBITION OF PATIENT.—DR. BEVAN.

The case before you is one of gastrostomy done for a stricture near the cardiac end of the stomach. The patient was transferred from the medical to the surgical side on December 20th. She had been in the hospital before for trouble of a specific character, and there remained well-marked evidences of it. The local indications calling for treatment were intense emaciation, which had been progressive and rapid with almost absolute inability to swallow. Examination with various probes showed an obstruction 13½ inches from the teeth, through which not even the smallest probe would pass; some fluid would pass. An operation to open the stomach and bring its edge up to the margin of the skin wound and feeding entirely through this was tried. The progress of the case has been uneventful except that she has put on flesh with great rapidity. She almost died on the table because of her great weakness, but is now able to walk about, and takes a great deal of active exercise. What is equally of importance is, that since the operation was performed she has become able to swallow some liquid food through, I suppose, the rest that has been given to the strictured tissue.

CASE 2. Popliteal Aneurism. This man entered the house last Monday, a week, giving the history of syphilis dating back some nine or ten years, for which he had received practically no treatment. Last May he detected a small lump in the popliteal space. In a short time this began to enlarge, and finally attained enormous size, so that when he came in there was an enormous mass occupying this whole space and destroying all normal relations about the knee-joint. The mass was semi-fluctuating and had an indistinct pulsation. There was no bruit, but the diagnosis was made of a ruptured popliteal aneurism, and acting on that, the incision which you see was made and the mass dissected out. The specimen will be passed around.

The sac had ruptured and the entire space was filled with blood-clot; I saw no free blood but a tourniquet had been very carefully applied. As rapidly as possible the incision was extended, a double ligature passed around the upper portion of the popliteal, and the sac was enucleated until the extreme limit of the dilated vessels was met with and then these were tied and the mass removed. We have thought it advisable to treat the wound as a local one. The temperature reached at one time 103, but is now below 100.

Popliteal aneurisms are of unusual occurrence. In an experience of many years I have met with but two in which the vessels had ruptured and the conditions were similar to this. They were treated in the same way, and I believe the method is preferable to that more commonly used, of amputation.

DISCUSSION.

*Dr. Randolph Winslow:* I was very much interested in Dr. Bevan's remarks and the cases which he has shown. I would like to ask Dr. Bevan whether, in the case of gastrostomy, the wound leaks, and whether, when food is put into the wound, any of it runs out.

*Dr. Bevan:* To a very slight extent, it does.

*Dr. Winslow:* I understood you to say you merely stitched the stomach wall to the integument and did not perform any of the other operative measures, such as suggested by Carter and others?

*Dr. Bevan:* No, there was no effort at valve formation.

*Dr. Winslow:* Was it the intention to have a permanent fistula or a temporary one?

*Dr. Bevan:* Merely a temporary one.

*Dr. Winslow:* Of course it depends entirely upon what the condition is due to, whether the trouble is malignant or whether it is a stricture of the esophagus, as I take this to have been. If the condition is a malignant one, then of course the operation is only performed after the patient is unable to swallow and the fistula is expected to be a permanent one. In that case, I think one of the methods of operation, which seeks to form a valve for the purpose of preventing regurgitation through the fistula, should be performed. If, on the contrary, the fistula is a temporary establishment only for nutrition until the patient is in condition for further operation, then the method adopted by Dr. Bevan is eminently proper. If it is to be permanent I do not think this method should be adopted, as it will certainly leak, cause excoriation of the skin, and the patient will be uncomfortable. It does give a better opportunity to attack the stricture by sounds from below or by means of the string saw. Then the rest, as Dr. Bevan has said, from the irritation of swallowing and of food passing over the inflamed tissue, is of vast benefit in these cases. It is probable that Dr. Bevan will have to resort to further procedures for the correction of the stricture that is present; such a procedure as passing graduated sounds or employment of a string by means of which the stricture is sawed so that instruments can be passed. I have operated a number of times, in all but one for cases of acute stricture, the result of corrosive poisoning. In one case I could not succeed in establishing continuity of the esophagus, and after a year the patient died. In another case, after feeding the child through the fistula for a long time I succeeded in overcoming the stricture, and sent the child home with a patent esophagus.

*Dr. Bevan:* I have nothing further to add to the discussion save in the way of explanation. The theory on which the operation was done was, that there existed a syphilitic stenosis near the cardiac end of the stomach, and the operation was intended merely for temporary effect and to afford the facilities for dilating from below upwards. With that end in view, the operation was made as high up on the stomach wall and as near the cardiac end as we could reasonably make it.

A CASE OF CHOREA IN AN ADULT.—DR. GEORGE J. PRESTON.

This case is a very unique one, in my experience, and one that is certainly very difficult to assign to its proper class. His history is briefly as follows: his father died of bronchial trouble, his mother in confinement, and one sister died of pulmonary tuberculosis. Personally he has had the usual diseases of childhood, and when about three years of age, and this may be of some importance, he was suddenly awakened from sleep by a fright, and choreic movements set in and have persisted ever since. You will see the peculiar movements about the face and head as he sits here and observe his peculiar gait as he walks. His speech is broken and indistinct and articulation very peculiar. There is no mental weakness and a physical examination reveals nothing abnormal. The muscular movement is tolerably constant except when he sleeps.

The interesting point in this case is the diagnosis. The movements are choreic, but certainly it is not a case of chorea vulgaris, the movements differing very much from that, nor is it a case of some of those peculiar forms of chorea, the fibrillary, the variety described by Dubini as occurring in Italy, nor the electrical chorea belonging perhaps to the class of ordinary habit choreas. In the last year or two I have seen several cases of this distinct habit chorea, and I have a most typical case now under my care, of a young girl who has had it the most of her life. The movements are electrical: she will be sitting quite still and all of a sudden there will come this quick, electric-like movement. Unfortunately that variety is incurable, and fortunately it is not very common. This case does not seem to belong to any of these forms, and it is a question whether it should be classed with the Huntingdon variety. About 25 years ago, Huntingdon described a class of choreas that were distinctly hereditary. His father and grandfathers had been practising on Long Island for many years and had observed a number of cases such as he described. It runs through from 2 to 5 generations, begins between 30 and 40 years of age and always has a certain amount of mental complication. The cases run a comparatively short course and finally terminate in dementia. This case began in early life, has been running for something like 30 years, and is not interfering with the man's mental condition at all. He had been able to do hard manual labor all his life and support a family of a wife and three children.

It does not seem to belong to the class sometimes described as chronic chorea, which is practically identical with the Huntingdon variety, nor to the class of cases known as athetosis, for in both of these there is usually marked mental deterioration. I have seen but one case anything like it, and that was a case of congenital chorea that I described a few years ago, where the movements were like these but more exaggerated. The movements of the tongue and facial muscles had been of such a nature that that patient had never learned to talk. She was very intelligent, particularly fond of music, learned to play the piano well and could understand everything that was said to her but was totally unable to express herself. This man has never been able to learn to write, though he went to school for five years and progressed as far as the grammar school.

In attempting to make a diagnosis the only thing we can say is that the case belongs to the chorea group. It emphasizes the fact that we cannot speak of chorea as a distinct disease with a clear onset of symptoms and a complete entity, but must consider it as a symptom complex, a set of symptoms with varying pathology and symptomatology but conforming to a certain general type. Then a case like this could be said to belong to that group, while we recognize the fact that none of the choreas have a distinct and decided pathology, but that all are symptoms complex of some general condition which must certainly be a disturbance of the motor cortex.

DISCUSSION.

*Dr. Hermon:* This case is very interesting from the diagnostic point of view, but it would also be interesting if Dr. Preston would tell us what therapeutic measures he has taken. There are certain cases which we cannot classify in a distinct group, especially in nervous diseases, and we then usually have recourse to the term hysteria. It is possible that this is one of the cases in which our inability to classify would lead us to take this course.

*Dr. Abercrombie:* What effect have acute affections had upon his condition?

*Dr. Preston:* He has had most of the acute diseases, practically all of those of childhood, and pneumonia, rheumatism and syphilis, all of them subsequent to the time the choreaform movements began.

In reply to Dr. Hermon's question, I would say that the man has not been long in the hospital, and no very definite measures have been tried as yet. He has been given some sedatives and medicines of that nature. The theory of hysteria occurred to me early, but as these symptoms have been going on for thirty years without any abatement, and seem to belong so certainly to the choreic group, I have thought it belonged to that class.

EXHIBITION OF SURGICAL CASES.—DR. J. W. CHAMBERS.

CASE 1. Strangulated Hernia. This patient was operated on two days ago. About four days ago, while splitting wood, he felt an acute pain in his abdomen and a lump appeared there. This was followed by a period of vomiting and nausea, and a physician who was called in from Havre de Grace sent him into the hospital. He arrived about eight o'clock Tuesday night, and his trouble was recognized as a strangulated hernia. On Wednesday morning the hernial sac was removed and was found to be a congenital one. I used the Murphy button in closing up the wound, and his recovery has been good. Stitches were used over the button to reinforce it, for I do not think it is the best means to employ, and should be better satisfied, when possible, to avoid the use of the button. I have had trouble with it at times, and in one case, although the patient did well for six days, it then caused ulceration and perforation. I think the time has about passed for the use of these mechanical methods, and I never employ them without rather wishing I had not.

CASE 2. A Fibro-adenoma of the Breast. I merely wish to show this specimen, which is a tumor of the breast removed from a child of seventeen. It is an immense tumor to remove from a child.

CASE 3. This man had a carcinoma removed from the breast eight years ago, and six weeks ago I removed another growth of the same character and exposing the pleura pretty thoroughly, but the patient within twenty-four hours was sitting up, and in three days was about the room and able to leave the hospital at the end of the week. This was against protest, of course, but the wound is practically well. At the operation the lung collapsed for a few moments, but soon filled up with air. I am inclined to think the second growth was independent of the first, as there was no evidence of metastases.

EXHIBITION OF MEDICAL CASES.—DR. CAREY GAMBLE.

I have here two cases of enlargement of the heart that are of considerable interest. The first man's history is as follows: He has led a pretty rough life and had three attacks of inflammatory rheumatism? In addition to that he has suffered with lues and several attacks of urethritis. Eighteen months ago he was taken with a feeling of pain and pressure about the breast, as though something were clutching him about the breast bone, with pain referred to the cardiac area. He was employed up to a year ago at hard laboring work, but lately he has not been able to do very much.

He came in January 1st with swelling of the face, hands and feet, and we found an interesting condition of the heart. It is a classical case of aortic regurgitation. You can see the pulsations in the neck and the wave that extends to the anterior axillary line. He has the Corrigan pulse and the capillary pulse is visible in the finger-nails. When the skin is scratched he has the alternate fading and blushing that is sometimes noted. Over the second space he has a distinct systolic and diastolic murmur, typical in tone and transmitted into the vessels of the neck. When he first came in there was obliteration of the second sound, but since he has been here the second sound has returned and is distinct in spite of the murmur.

CASE 2. This man has not given us any history of angina-like pain, and the only history of illness we can get is that he had an attack of inflammatory rheumatism four years ago, from which he recovered after a continuous illness of six months. He went for a short time on an oyster boat where he developed pain about the breast, cough and swelling of the legs and abdomen. The murmurs are much more complicated than in the other case. He has a thrill distinctly localized and presystolic in time. In the second interspace there is a diastolic and systolic murmur transmitted to the left and a systolic regurgitating murmur towards the axilla. He also has the Corrigan pulse. From his anemia we thought probably he had involvement of the kidney, but we have not been able to find either casts or albumen in the urine. Probably his life on the Bay with hard labor and poor food had much to do with his condition at the time he came in. The provisional diagnosis in this case is that he had first an aortic regurgitation and probably a stenosis and regurgitation of the mitral valve.

DISCUSSION.

*Dr. Henkel:* I would like to ask what the treatment has been in these cases?

*Dr. Gamble:* Principally, rest. They have been kept flat on their backs and both were given small doses of infusion of digitalis. It seems to have had a good effect. The three things they have had is rest in bed, digitalis and diet.

EXHIBITION OF SURGICAL CASES.—DR. I. R. TRIMBLE.

CASE 1. I bring this case simply to show the result of quite a large abdominal incision. Six years ago he was run over by a wagon and was laid up in bed for two months, after which he went back to work at street-paving. On the 5th of June last, after an attack of acute pain in the side, he came to the hospital with a distinct fluctuating mass that was made out at the time to be a liver abscess. He refused to be operated upon then but returned June 30th, and an extensive incision was made running down below the anterior-superior spine of the ilium. He was in very bad shape, and we packed the wound and made a free opening into the liver and another in the gall-bladder. He made an uneventful recovery except that we cannot find any liver dullness on the right side at all. The large abscess had left the right lobe simply a shell.

CASE 2. Three months ago this child had typhoid fever, followed by pneumonia and pleurisy, and I show him with an enormously dilated left chest and dullness up to the second rib. He seemed to be dying of empyema. At the operation we resected two ribs to allow free drainage, and he has gotten along very well, as you see. The lung is expanding and coming, as you can determine by percussion. We did not irrigate in this case.

CASE 3. This is an interesting case in two or three different ways. The man is 73 years old. When a young boy about seventeen he was thrown from a horse and a double direct inguinal hernia resulted. Four weeks ago he fell down-stairs, and when he got up was unable to bend his knee. Examination showed that he had ruptured the quadriceps extensor. It is the only case of the kind I have ever seen. A $\frac{1}{2}$ per cent. solution of cocain was injected and the muscle was stitched to the tendon tissue over the knee cap. I have not tried movement yet but hope for a good result.

I want to call attention to the action of cocain in this case. We estimated that we got about one half grain under the skin, but before he left the table he was delirious, with a pulse of 160 and respirations 45 per minute, and we feared he would die. From the time the cocain was injected until the knife laid bare the tissues certainly was not more than three minutes.

CASE 4. This man is a B. and O. brakeman, who, nine weeks before I saw him, received a backward dislocation of the hip. When he came in he moved about with difficulty. A physician had reduced the dislocation, as he thought, but had really dislocated it again forward over the pubes.

The bone was gotten back with some difficulty, he was put up in a long splint, and at the end of three weeks was walking around as you see.

Dr. Harvey G. Beck exhibited a rare case of monstrosity. (Published in *Maryland Medical Journal* September, 1900.) The specimen show a number of abnormalities, such as hair-lip, cleft palate acrania, hydrencephalocele and spina bifida. The child was a still-born female weighing 219 grams and measuring 27 cm. in length by 24 cm. in circumference of the body. The mother was 25 years of age and her two former labors were of normal character. No history could be ascertained that would suggest an explanation for the monster.

Dr. Keirle exhibited a number of interesting pathological specimens.

A smoker was held at the close of the meeting.

Adjournment. H. O. REIK, M.D.,
5 W. Preston St.

---

### SUBCUTANEOUS SALINE INJECTIONS IN PNEUMONIA.

Penrose's statistics as to the favorable effect of the subcutaneous injection of normal salt solution in pneumonia were so favorable that the method was tried by Drs. Ewart and Beaumont in 6 cases under their care. The cases selected were those occurring in unhealthy and debilitated persons, where the disease seemed likely to be severe. The first case was of a severe type in a debilitated female, aged thirty-seven years. One pint of the solution was injected into the subcutaneous tissue of the right subclavian region. Next day the same amount was injected into a similar situation on the left side at 11 a. m., and again at 6 p. m. Altogether there were four such injections alternately on the left and right sides. The patient recovered. The other 5 cases, 1 of which was ordinary pneumonia, 2 double, 1 complicated with empyema, and 1 case of pneumo-typhoid fever, all died. Grey hepatization had appeared in some. The results were disappointing. All but 1 were fatal, but it might be said that the proceeding caused no unfavorable symptoms, and in one or two seemed to have delayed the fatal issue. It did no further good, neither did it promote death. It was not resented by the patients, and the reporters thought it might be given a further trial.

